# Supplementary material for: A Monoallelic Variant in REST Is Associated with Non-Syndromic Autosomal Dominant Hearing Impairment in a South African Family
Source: Genes (Basel). 2021 Nov 6;12(11):1765. doi: 10.3390/genes12111765 (PMC8618167; doi:10.3390/genes12111765)
Supplement: Supplementary file 1 [file genes-12-01765-s001.zip › genes-1429965-supplementary.pdf]

Supplementary material

Table S1: Variations Identified using whole-exome sequencing in Family 1

Gene	Refseq ID	Variation	Protein consequence	Inheritance model	Homozygous/ Heterozygous	Known/Novel variant	ACMG Classification	Verdict
CDH23	NM_022124.5	c.5653C>T	p.(R1885C)	Autosomal recessive	Heterozygous	Known	PM1_M, PM2_M, PP2_P, PP3_P	LP
NDUFAF3	NM_199069.2	c.188dupA	p.(Y63*)	Autosomal recessive	Heterozygous	Known	PVS1_VS, PM2_M, PP3_P	P
REST	NM_005612.4	c.1244G>C	p.(C415S)	Autosomal Dominant	Heterozygous	Novel	PM1_M, PM2_M, PP1_P	VUS

xxx_VS = Very Strong; xxx_M = Moderate; xxx_P = Supportive; P = Pathogenic; LP = Likely Pathogenic; VUS = Variant of uncertain significance

Table S2: Demographic and phenotypic of individuals of sporadic HI of putative genetic origin

Statistic		Frequency	Percentage
Sex	Male	22	42,3
	Female	30	57,7
Age	Median	15	
	Mean	18	
Ethnicity	Black	43	82,7
	Mixed Ancestry	9	17,3
Mechanism of HI	Sensorineural	45	86,5
	Mixed HI	7	13,5
Localisation of HI	Unilateral	0	0,0
	Bilateral	52	100,0
	Unspecified	0	0,0
Degree of HI Right	No Impairment	0	0,0
	Mild	0	0,0
	Moderate	0	0,0
	Severe	4	7,7
	Profound	45	86,5
	Unspecified	3	5,8
Degree of HI Left	No Impairment	0	0,0
	Mild	1	1,9
	Moderate	0	0,0
	Severe	7	13,5
	Profound	40	76,9
	Unspecified	4	7,7
Onset of HI	Congenital	31	59,6
	Perilingual	11	21,2
	Postlingual	7	13,5
	Unspecified	3	5,8

Table S3: Demographic information of the control population

Statistic		Frequency	Percentage
Gender	Female	78	75.7
	Male	25	24.3
Age	Mean	55.7	
	Median	58	
Ethnicity	Black	103	100.0
	Mixed Ancestry	0	0.0

Table S4: Functional prediction for the candidate variant identified in *REST*

Prediction Tool	c.1244G>C
CADD	11.26
DANN	0.892
dbSNP rs number	Absent
Eigen	0.374
Eigen-PC	0.31
FATHMM	Tolerated
FATHMM-MKL	Damaging
Frequency in gnomAD	0
GERP++	4.96
LRT	Deleterious
M-CAP	Tolerated
MetaLR	Tolerated
MetaSVM	Tolerated

MutationAssessor	Low
MutationTaster	Disease Causing
MutPred	0.308
PhastCons	1
PhyloP	9.504
Polyphen2 HDIV prediction	Probably Damaging
Polyphen2 HVAR prediction	Probably Damaging
Predicted effect	Missense
PROVEAN	Damaging
REVEL	0.354
SIFT	Tolerated
SiPhy	14.723

RefSeq used: NM_005612.5

Figure S1: Schematic Diagram of REST, isoform 1, with some of its transcriptional co-repressors interacting with the repressor domains

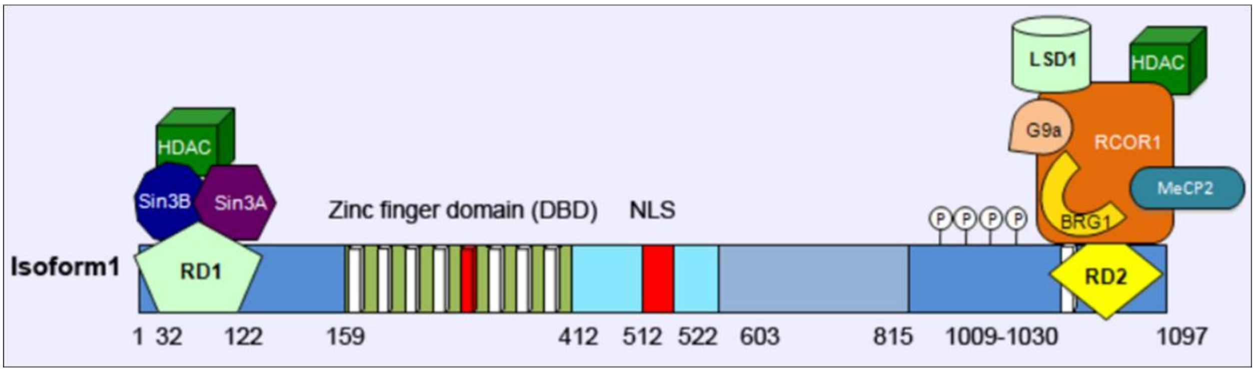

The protein consists of 2 repressor domains (RD1 and RD2); one at the amino terminal and one at the carboxyl-terminal of the protein. It has 8 zinc fingers in the DNA binding domain (DBD) and two nuclear localization domains (shown in red; of which one is a zinc finger). REST has a ninth zinc finger close to RD2 and has a lysine-rich domain (from position 400 to 603) and a proline-rich domain (from position 595 to position 815). Adapted from the Atlas of Genetics and Cytogenetics in Oncology and Haematology

Figure S2: REST secondary structure prediction by the PSIPRED 4.0 workbench.

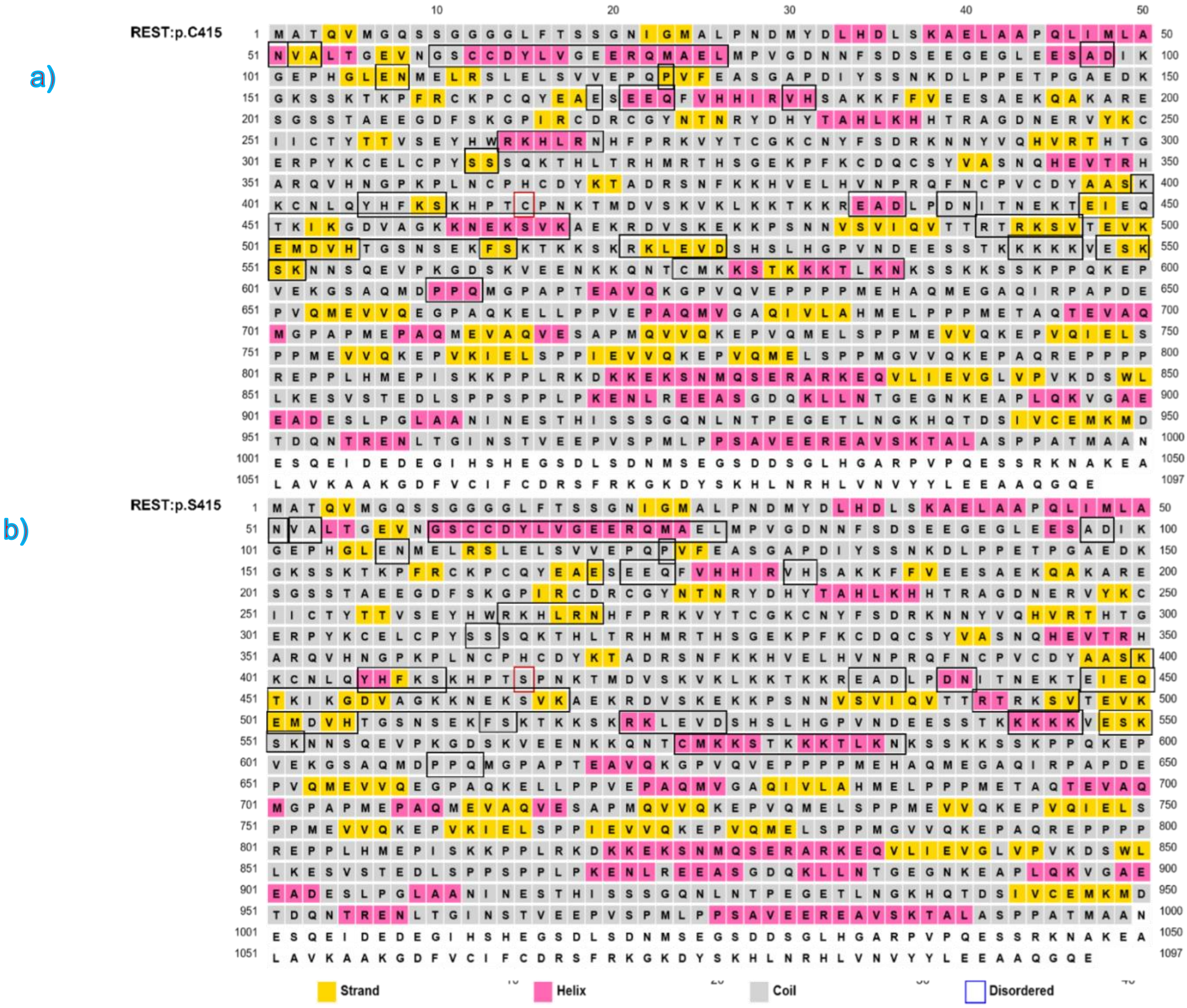

Yellow regions represent beta strands, pink regions represent alpha helices, grey regions represent coils, while plain regions (no colours) represent areas of disorder. Black and red boxes indicate positions of difference between the wild type (REST:p.C415) and mutant (REST:p.S415) proteins. The red box shows the mutant site (C415S), while the black boxes show loss of, and attenuation of α helices and β strands in the mutant protein as compared to the wild type protein.

Figure S3: Disulphide bond order in WT REST and C415S REST

a)

Predicted bonds	
62 - 63	EVNGSCCDYLV – VNGSCCDYLVG
161 - 278	TKPFRCKPCQY – RKVYTCGKCNY
164 - 306	FRCKPCQYEAE – ERPYKCELCPY
218 - 250	KGPIRCDRCGY – ERVYKCIICTY
221 - 334	IRCDRCGYNTN – EKPFKCDQCSY
253 - 337	YKCIICTYTTV – FKCDQCSYVAS
281 - 391	YTCGKCNYFSD – PRQFNCPVCDY
309 - 945	YKCELCPYSSS – TDSIVCEMKMD
363 - 402	PKPLNCPHCDY – AASKKCNLQYH
366 - 394	LNCPHCDYKTA – FNCPVCDYAAS
415 - 1062	SKHPTCPNKTM – KGDFVCIFCDR
574 - 1065	KKQNTCMKKST – FVCIFCDRSFR

b)

Predicted bonds	
62 - 402	EVNGSCCDYLV – AASKKCNLQYH
63 - 278	VNGSCCDYLVG – RKVYTCGKCNY
161 - 250	TKPFRCKPCQY – ERVYKCIICTY
164 - 218	FRCKPCQYEAE – KGPIRCDRCGY
221 - 306	IRCDRCGYNTN – ERPYKCELCPY
253 - 394	YKCIICTYTTV – FNCPVCDYAAS
281 - 334	YTCGKCNYFSD – EKPFKCDQCSY
309 - 391	YKCELCPYSSS – PRQFNCPVCDY
337 - 363	FKCDQCSYVAS – PKPLNCPHCDY
366 - 945	LNCPHCDYKTA – TDSIVCEMKMD
574 - 1065	KKQNTCMKKST – FVCIFCDRSFR

The shift in disulphide bonds is indicated with the WT represented in A) and the MT represented in B). the mutation results in far-reaching changes in the disulphide bond formation with only the bond between cysteines 574 and 1065 being preserved between the WT and MT proteins.
